# Congenital Zika virus infection in laboratory animals: a comparative review highlights translational studies on the maternal-foetal interface

**DOI:** 10.1590/0074-02760240125

**Published:** 2025-02-28

**Authors:** Noemi Rovaris Gardinali, Renato Sergio Marchevsky, Yara Cavalcante Vieira, Marcelo Pelajo-Machado, Tatiana Kugelmeier, Juliana Gil Melgaço, Márcio Pinto Castro, Jaqueline Mendes de Oliveira, Marcelo Alves Pinto

**Affiliations:** 1 Fundação Oswaldo Cruz-Fiocruz, Instituto Oswaldo Cruz, Laboratório de Desenvolvimento Tecnológico em Virologia, Rio de Janeiro, RJ, Brasil; 2 Fundação Oswaldo Cruz-Fiocruz, Instituto de Tecnologia em Imunobiológicos, Bio-Manguinhos, Laboratório de Tecnologia Virológica, Rio de Janeiro, RJ, Brasil; 3 Fundação Oswaldo Cruz-Fiocruz, Instituto de Tecnologia em Imunobiológicos, Bio-Manguinhos, Departamento de Experimentos Pré-Clínicos, Laboratório de Ensaios Pré-Clínicos, Rio de Janeiro, RJ, Brasil; 4 Fundação Oswaldo Cruz-Fiocruz, Instituto Oswaldo Cruz, Laboratório de Medicina Experimental e Saúde, Rio de Janeiro, RJ, Brasil; 5 Fundação Oswaldo Cruz-Fiocruz, Instituto de Ciência e Tecnologia em Biomodelos, Rio de Janeiro, RJ, Brasil; 6 Fundação Oswaldo Cruz-Fiocruz, Instituto de Tecnologia em Imunobiológicos, Bio-Manguinhos, Departamento de Experimentos Pré-Clínicos, Laboratório de Tecnologia Imunológica, Rio de Janeiro, RJ, Brasil; 7 Centro de Diagnóstico Veterinário, Niterói, RJ, Brasil; 8 The Pennsylvania State University, Department of Food Science, University Park, PA, USA

**Keywords:** Zika virus infection, vertical transmission, teratogenesis, haemochorial placenta, maternal-foetal interface, nonhuman primates, AG129 mice, sofosbuvir, antiviral drugs

## Abstract

The 2015-16 Zika virus (ZIKV) epidemic has posed unprecedented concern for maternal-infant health, mainly due to the substantial risk of microcephaly and other neurological birth abnormalities associated with congenital ZIKV syndrome (CZS). As licenced vaccines and effective antivirals are still unavailable, attention has been focused on post-delivery *in vitro* or translational *in vivo* studies to understand the impact of maternal ZIKV infection on placentation and neurodevelopmental consequences for the foetus. Here, we review clinical and translational studies highlighting ZIKV-induced maternal-foetal interface dysfunction, adding to our previous observations of experimental ZIKV vertical transmission to pregnant rhesus monkeys and newly published post-epidemic findings about the theme. This comparative review focuses on the mechanisms by which the virus has a cytopathic effect on trophoblasts and macrophages during placentation in humans, nonhuman primates, and rodent transgenic models, crosses the placental barrier, replicates, and establishes a persistent uteroplacental infection. When considering the mechanism of ZIKV-induced birth defects in humans and other susceptible hosts, it becomes apparent how the various stages of the ZIKV cycle in the host (both the parent and offspring) unfold. This understanding presents specific opportunities for pharmacological intervention and the development of preventative vaccines.

Historical and epidemiologic aspects of ZIKV infection

Zika virus (ZIKV), a mosquito-borne flavivirus whose vectors are *Aedes aegypti* and *Aedes albopictus*, is widely disseminated in Brazil^1^ and can infect humans and nonhuman primates. Indeed, ZIKV was first isolated from a rhesus monkey in 1947 and *Aedes africanus* in 1948.^2^ In humans, the infection was first described in Nigeria, Africa.^3^ Ordinarily, ZIKV infections have been reported to be sporadic and commonly associated with mild disease. The first well-known outbreak of the ZIKV occurred in the Yap Islands of Micronesia in 2007. According to local public health reports, the Zika infection rate ranged from 73%,^4^ the outbreak of French Polynesia in 2013-14 followed by general prevalence rates of 49% with an estimated 32,000 infected patients between October 2013 and April 2014.^5^ ^,^ ^6^ In the Brazilian outbreak, neurological disorders such as Guillain-Barré syndrome in adults reported an incidence between April-July 2015 among those ≥ 12 years of age was 5.6 cases/100,000 population/year and increased markedly with increasing age to 14.7 cases/100,000 among those ≥ 60 years of age.^7^ An effective link between the vertical transmission of the ZIKV and microcephaly emerged from the epidemic in Brazil in 2015-2016, with infection rates estimated to range from 10% to 80%.^8^ ^,^ ^9^ ^,^ ^10^ Earlier, during the 2013-2014 Zika outbreak in French Polynesia, the risk of microcephaly related to ZIKV infection was 0.95%, as estimated from eight cases identified retrospectively in the population of 270,000 inhabitants, with a 66% ZIKV infection rate.^11^ ^,^ ^12^ In contrast, the Brazilian Live Births Information System reported thousands of microcephaly or other neurodevelopmental-associated anomalies between July 2015 and February 2016.^13^ ^,^ ^14^ The Brazilian ZIKV outbreak, two distinct waves of infection, extended across all Brazilian regions in 2015 and 2016. 1,673 272 cases were notified, of which 41,473 (2-5%) were in pregnant women. During the most severe first wave, 1950 cases of microcephaly were confirmed [1373 (70.4%0] occurred in the Northeast region, with a peak monthly estimated at 49.9 cases per 10,000 live births.^15^ In the Brazilian outbreak, approximately one-third of liveborn children with prenatal ZIKV exposure presented with at least one abnormality compatible with congenital infection, while the risk of presenting with at least two abnormalities in combination was less than 1.0%.^16^

The spectrum of sequelae of congenital ZIKV infection, broadly referred to as congenital ZIKV syndrome (CZS), in which microcephaly is a major manifestation, includes (but is not limited to) parenchymal or cerebellar calcification, ventriculomegaly, hydrocephalus, arthrogryposis, and visual and hearing disorders.^17^ ZIKV infection during pregnancy can be associated with neurodevelopmental abnormalities, even in normocephalic infants or those born to asymptomatic mothers. Neurodevelopmental outcomes possibly associated with congenital ZIKV infections include shorter attention spans, longer processing times of visual stimuli, postnatal microcephaly, lissencephaly, hypotonia, hypertonia, arthrogryposis, hearing tissues (inner ear), transient developmental delay, delayed myelination, persistent intracranial calcifications, ventriculomegaly, cerebellar hypoplasia, and cortical abnormalities, as revised by Caldwell and colleagues.^18^ Early identification and intervention for neurodevelopmental disorders can improve cognitive, social, and behavioural functioning.^19^ The objectives of this manuscript were to compare ZIKV-induced maternal-foetal interface changes in different laboratory animal species, highlight the differences among ZIKV vertical transmission in the light of anatomical differences in placentation, and add some unpublished results of our previous study evolving ZIKV infection at the early third of prepregnancy of rhesus monkeys and its vertical viral transmission.

Relevance of the maternal-foetal interface for ZIKV vertical transmission

Physiologically, during early human pregnancy, the uterine mucosa transforms into the decidua, into which the foetal placenta implants and where placental trophoblast cells and maternal cells intermingle and communicate.^20^ In discoid placentation, maternal and foetal circulations are interwoven, with trophoblast and endothelial cell layers differently organised depending on the species: haemomonochorial in humans and haemotrichorial in mice, which represents an additional endothelial barrier to protect the foetus from invading pathogens. Another similarity between the human and NHP species that may contribute to foetal infection is the long gestational period of Old-World monkeys (150 to 210 days) and human beings (280 days), in contrast with rodents (20 days).^21^ ^,^ ^22^ As estimated from translational studies with rhesus monkeys, the viraemia period of ZIKV-infected (at the first gestational trimester) dams lasts 7 to 10 days;^23^ such prolonged maternal viraemia can trigger transplacental viral transposition, virus crossing into the amniotic fluid (AF), and in utero foetal death.^24^ The ZIKV strain infects and replicates in primary human placental macrophages (Hofbauer cells) and poorly in cytotrophoblasts. Viral replication induces type I interferon (IFN), proinflammatory cytokines, and antiviral gene expression but causes minimal cell death. In addition to suggesting a mechanism for intrauterine transmission in which ZIKV gains access to the foetal compartment by directly infecting placental cells and disrupting the placental barrier,^25^ other authors have suggested that ZIKV can open the paracellular pathway of STB cells.^26^ In human ZIKV vertical transmission, monocytes/macrophages (Hofbauer cells) have been referred to as “Trojan horses” since they may carry infectious virus particles to immune-privileged sites such as the placental, blood-testis, and blood-brain barriers.^27^ Another hypothesis is that low-affinity circulating maternal antibodies could enhance ZIKV replication by binding to Fc receptor (FcR)-bearing cells via antibody-dependent infection enhancement (ADE), *i.e.*, cross-reactive antibodies (against other flaviviruses) transported transplacentally via neonatal FcRs, which could improve ZIKV replication in pregnancy-associated progenitor cells (PAPCs).^28^ The prolonged period of ZIKV viremia observed in pregnant macaques and human beings has been justified by the hypothesis that the infected foetus and/or placenta role as a ZIKV reservoir that cannot be efficiently cleared by the dam immune system however, this hypothesis needs to be best investigated.^22^

Together, observations in NHPs and humans indicate that foetal abnormalities caused by ZIKV infection can occur regardless of maternal signs. A Brazilian epidemiological report reinforced that previous dengue fever epidemics may be related to microcephaly incidence and the idea of a window of cross-protection and a window of increased risk.^29^ This is a controversial issue, as an experimental study using a pregnant rhesus monkey did not reveal foetal abnormalities at delivery; however, more ZIKV RNA was detected in the placenta of macaques immunised to DENV, suggesting that DENV immunity could enhance ZIKV infection of the placenta.^30^

ZIKV infection in the circulation of pregnant women and macaques crosses the placental barrier (endothelial cells and trophoblasts), and trophoblasts represent the first viral replication site in the foetus, since primary trophoblasts express ZIKV cell entry receptors. There are controversies about whether ZIKV-infected trophoblast function may be preserved and become a compartment of foetal viral dissemination.^31^ As demonstrated in dizygotic twins, which present different outcomes after infection with a Brazilian isolate of ZIKV, trophoblasts from the ZIKV-nonaffected twin secreted increased levels of inflammatory chemokines (RANTES/CCL5 and IP10) because the nonaffected twin more efficiently activated genes (induced by IFN-g) involved in placental ZIKV immune protection, thus preventing ZIKV dissemination into developing foetus tissues.^32^ Another report about discordant outcomes in dizygotic twins in Brazil is justified by gene mutations in the *MTOR* and Wnt pathways, which regulate foetal neurodevelopment.^33^ ^,^ ^34^ Indeed, foetal protection requires the induction of a robust placental antiviral response, with type I IFN binding to the IFN alpha receptor (IFNAR) expressed in many cell types and upregulating IFN-stimulated genes (ISGs) in the placenta.^35^ ZIKV single-stranded RNA elicits an antiviral response via receptor acid-inducible gene I (RIG-I), resulting in robust type I and III IFN responses.^36^ The decidua microenvironment is physiologically tolerogenic.^37^ During ZIKV infection, dNK cells (the main immune cell population of the first-trimester decidua) produce proinflammatory cytokines that may regulate ZIKV infection during pregnancy.^38^ On the other hand, the immune system recruit neutrophils, natural killer cells (dNKs), macrophages, T cells and dendritic cells (DCs) in the stromal decidua microenvironment, which alters the decidua, possibly leading to placenta immune-mediated injuries. The expression of IFN type I diminished extravillous trophoblast invasion by the spiral artery (EVT-mediated spiral artery remodelling), elevating the risk of hypertension and preeclampsia in pregnant women^39^ and pregnant rhesus monkeys.^23^ Additionally, in a type I IFN receptor-deficient (IFNAR-/-) mouse model, placental ZIKV infection can cause indirect damage to the foetus due to reduced uteroplacental perfusion, leading to intrauterine foetal growth delay and early embryonic death.^40^

Foetal neuropathologic findings associated with congenital ZIKV infection in animal models

Congenital ZIKV syndrome, described in the early Brazilian epidemic,^41^ has been extensively investigated through translational studies using immunodeficient mice (AG129)^42^ and immunocompetent pregnant nonhuman primates.^23^ ^,^ ^43^ ^,^ ^44^ Preclinical studies have confirmed that foetal neural progenitor cells (NPCs) are the principal target cells contributing to abnormal brain embryo development, as observed in ZIKV-infected pregnant mice and rhesus monkeys. In addition to pathogenic mechanisms, animal models are valuable tools for investigating the efficacy of repositioned or new antiviral drugs.^45^ *In vitro* studies have also been performed to understand the mechanisms underlying neuropathologic findings and the cytopathic effects of ZIKV on the death of NPCs and optic progenitor cells (OPCs).^46^ ^,^ ^47^ Other *in vitro* studies confirmed that the ZIKV E (envelope) protein causes cell cycle arrest, a decrease in cell proliferation, and an increase in the mitotic length of dividing human NPCs through dysregulating the cyclinD1-p21-p53 pathway and changing intercellular calcium levels and ATP stimulation in ZIKV E protein-expressing hNPCs.^48^ The direct neurotoxicity of the ZIKV envelope protein may be explained by the overexpression of polyadenosine diphosphate-ribose polymerase 1 in hNPCs.^49^ Another hypothesis is that ZIKV replication in the central nervous system (CNS) leads to direct cellular injury by increasing cellular glycolysis, which may cause intracellular stress and endoplasmic reticulum dysfunction^50^ associated with neuroinflammationm.^51^ AXL, a phosphatidylserine (PS) receptor, has been proposed to play a key role as an attachment factor for ZIKV infection in the host cell.^52^ However, genetic ablation of AXL does not affect ZIKV entry or ZIKV-mediated cell death in human induced pluripotent stem cell (iPSC)-derived NPCs or cerebral organoids.^53^ ZIKV tropism for glial cells is facilitated by the expression of the receptor tyrosine kinase (RTK) AXL, which impacts the transcriptional induction of type I IFN, proinflammatory cytokines and chemokines in infected NPCs, thus stimulating ZIKV replication.^54^ Consequently, this process results in the downregulation of foetal neurogenesis and the upregulation of NPC apoptosis, disrupting NPC cell cycle proliferation/self-renewal, which is essential for normal mammalian brain development.^55^ Other flaviviruses may also enter the CNS, causing peripheral neuropathy, including West Nile virus (WNV), Japanese encephalitis virus (JEV), and tick-borne encephalitis virus (TBEV).^18^ However, the neurotropic flaviviruses, which have direct cytolytic/oncolytic effects on NPCs, have shown beneficial effects on preclinical GBM models by killing glioma stem cells (GSCs).^56^ A 43-year-old woman was diagnosed with glioblastoma after the tumour resection was infected with the ZIKV outbreak in Brazil. Following the infection resolution, the glioblastoma regressed, and no recurrence was observed.^57^

ZIKV-induced inflammatory and vascular placental injuries alter the “immune clock” of pregnancy

The “immune clock of pregnancy” refers to dynamic changes in gestational immune status, which are chronologically necessary to maintain maternal immune homeostasis, thus supporting foetal growth while protecting the mother and foetus against infectious agents. Healthy-term pregnancy outcomes require synchronization of implantation competency by the blastocyst with endometrial receptivity. This short-term period of crosstalk between the blastocyst and the uterus, the so-called “window of implantation”, is controlled not only by ovarian oestrogen and progesterone hormones^58^ but by signalling molecules, including cytokines, growth factors, homeobox transcription factors, lipid mediators and morphogen genes, which function through autocrine, paracrine and juxtracrine interactions to support the effectiveness of blastocyst implantation.^59^ The interference of ZIKV and other congenital infections at the foetal-maternal interface in terms of the local cellular mechanisms that control maternal immune tolerance may help understand the teratogenesis mechanism.^60^

In a preclinical study of Asian-lineage ZIKV vertical transmission in pregnant rhesus monkeys, we confirmed the exacerbated placental anergic/suppressive mechanism of myeloid-derived suppressor cells (MDSCs),^23^ as suggested by Asian-lineage ZIKV infection of pregnant women’s blood leading to exacerbated M2-skewed immunosuppression of nonclassical monocytes in conjunction with global suppression of the type I interferon signalling pathway and aberrant expression of host genes associated with pregnancy complications.^61^ The phenotype of MDSCs was more frequent in placental cells from ZIKV-infected-Sofosbuvir(SOF)-treated dams, as well as in those from the negative control group, than in those from the ZIKV-infected-nontreated group. Not by chance, placentas from non-SOF-treated dams contained more of the viral replicative marker, anti-double-stranded RNA (dsRNA)-marked cells than those from SOF-treated dams. In both the untreated and SOF-treated groups, Hofbauer cells (CD3- HLADR- CD4+ CD14+) and T lymphocytes (CD3+) were detected in the placenta,^23^ confirming that the placental anergic/suppressive mechanism works by controlling viral-induced placental inflammation and preventing foetal death, as previously described by others. In two pregnant women with ZIKV infection, amniotic fluid collected by amniocentesis at 20 weeks gestation was RNA negative, and ZIKV RNA remained positive in the dam’s plasma until delivery, but the virus was not transmitted to the newborns. Similarly, two cases of maternal immunocompromised status were associated with a prolonged ZIKV viremia period.^62^

Non-invasive *in vivo* imaging studies have shown robust maternal-placental-foetal inflammation (chronic decidualitis, lymphoplasmacytic infiltrates, and neutrophilic leukocytoclastic vasculitis associated with spiral artery placenta) and calcification during ZIKV infection. Such uterine vasculitis affects the oxygen permeability of the placental villus, resulting in a significant decrease in oxygen delivery to the foetus.^63^ Similarly, in immunocompromised pregnant IFNAR-/- mice infected with ZIKV, placental inflammation was associated with impaired blood flow circulation in uteroplacental and foetal brain vessels, as revealed by colour Doppler ultrasound examination as early as 12.5 embryonic days, leading to intrauterine growth delay.^40^ Semmes and Coyne attributed severe ZIKV-induced placental inflammation in mice to a direct innate antiviral response^64^ and consequent foetal growth restriction or death due to blood supply restriction caused by microvascular disruption.^65^ Similarly, Pomar and colleagues described reduced umbilical artery blood flow and placentomegaly (placental thickness > 40 mm) in pregnant women with congenital ZIKV infection.^66^

The placental microenvironmental inflammation induced by ZIKV infection may also involve pericyte activation. Despite the poor description of ZKV infection, placental pericytes are essential for endothelial cell proliferation and placental microvasculature development. They surround the abluminal surface of capillary blood vessels through contractile cytoplasmic extensions that wrap around the endothelial cells lining the capillaries and venules. These antigen-presenting cells (APCs) play an essential role through contact-mediated and paracrine crosstalk with endothelial cells, and virus-infected pericytes may enhance viral penetration of the placenta and foetal blood-brain barrier, leading to neuroinflammatory and developmental sequelae in the foetus.^67^ Mouse models confirmed that ZIKV replication in pericytes from the choroid plexus impaired the permeability of the endothelial barrier and tissue perfusion.^68^ Similarly, human cytomegalovirus (HCMV) infection of the brain and retinal pericytes *in vitro* induces HCMV-associated neuroinflammation, reduced perfusion, and retinal dysfunction in neonates.^69^ In addition, the interaction of HCMV with pericytes in the placental microvasculature results in lytic infection. However, further studies investigating the infectivity of ZIKV in human placental pericytes and its potential role in ZIKV dissemination or the mechanisms of placental and foetal inflammation are needed.

Propositions to prevent congenital Zika syndrome

Translational studies using the rodent model interferon-α/β knockout mice (AG129) have highlighted the protective role of endogenous type I IFN against ZIKV infection,^42^ ^,^ ^70^ while other studies have reinforced the importance of secreted IFN-I by trophoblastic cells, which represents a possible mediator of pregnancy complications, including spontaneous abortions and growth restriction, in the context of congenital ZIKV infection.^71^ Classically associated with viral infections, type I IFNs activate downstream signalling pathways that increase the transcription of IFN-stimulated genes in the microenvironment, inhibiting viral replication and limiting viral spread.^72^ For example, ZIKV RNA activates Toll-like receptor-3 (TLR-3),^73^ dependent on Toll/interleukin-1 receptor/resistance protein (TIR).^74^ In addition, retinoic acid-inducible gene-I (RIG-I) mediates the innate immune response, triggering IFN production and antiviral actions to control ZIKV infection and placental inflammation.^75^

Sofosbuvir is a nucleotide analogue prodrug successfully used to treat chronic hepatitis caused by the hepatitis C virus (HCV), a Flaviviridae family member like the ZIKV. Its intracellular active triphosphate form inhibits HCV RNA polymerase and replication.^76^ ^,^ ^77^ The HCV and ZIKV RdRp-encoding genes are in the NS5B and NS5 domains. These genes are highly conserved among Flaviviridae members.^78^ The antiviral activity of SOF against ZIKV replication was demonstrated *in vitro*.^79^ ^,^ ^80^ Additionally, preclinical studies have confirmed its protective effect against ZIKV-induced teratogenesis in macaques.^23^ ^,^ ^81^ In addition, for the prevention of ZIKV vertical transmission and associated CZS, SOFs had less severe RNAaemia than did non-SOFs. The offspring of nontreated dams presented ZIKV neurotropism, whereas those of SOF-treated dams did not, suggesting an antiviral protective effect. At delivery, the offspring of nontreated dams exhibited macroscopic neural abnormalities such as lissencephaly, ventriculomegaly, and subdural haematoma (Figure, Table). Histological analysis revealed marked disorganisation of the cerebellar white matter folium, fold layer oedema, and ischaemic damage to the Purkinje and granular layers.^23^ The antiviral efficacy of SOF was confirmed to reduce ZIKV replication and prevent long-term sequelae in AG129 mice.^82^ In another SOF-treated rodent model, NOD/SCID mice, intravenous ZIKV infection prevented vertical transmission. Ouabain (an inhibitor of Na+, K+-ATPase) has been shown to act as an anti-ZIKV replication drug by binding to the ZIKV NS5-RdRp and NS3-helicase proteins^83^ and in a mouse model of CZS.^84^ The macrolide antibiotic azithromycin (AZM) reduces ZIKV proliferation and has cytoprotective effects on glial cell lines and human astrocytes.^85^ AZM upregulates the expression of host type I and III interferons and several downstream interferon-stimulated genes (ISGs) in response to ZIKV infection.^86^ No new clinical studies have been published to date, and other *in vitro* studies on anti-flavivirus agents have been published.^87^

**Figure:**
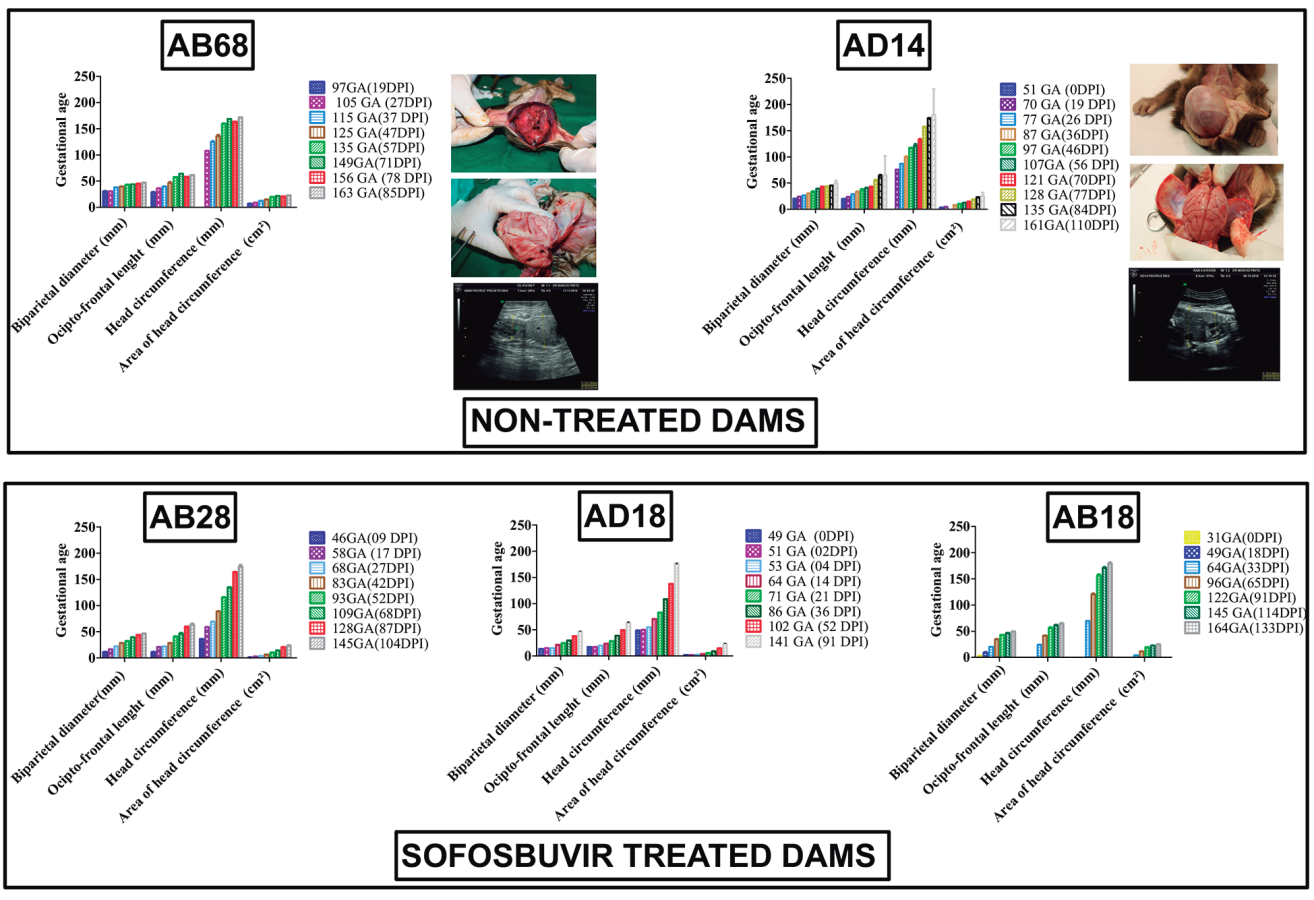
Gestational ultrasonographic follow-up showing foetal protection in pregnant rhesus monkeys inoculated with Zika virus (ZIKV) and treated with or without sofosbuvir (SOF). The parameters adopted included the biparietal diameter, occiput-frontal length, head circumference, area of head circumference and thorax constriction. AB68 offspring showing postnatal congenital ZIKV-induced injury. AD14 offspring exhibiting normal development.^23^

**TABLE:** Summary of clinical and virological findings of offspring from Zika virus (ZIKV)-infected macaque dams treated or not with sofosbuvir (SOF) as previously reported by Gardinali and colleagues^23^

Offspring identification	SOF treatment	Delivery GA/dpi	Foetal defects	*Birth weight	IgM	IgG	PRNT	ZIKV RNA detection log_10_		
								Amniotic fluid (log_10_/dpi)	Placenta	Foetus
AD14	No	Spontaneous 160/109	No	500	0.188	1,196	31,250	2.9/56 ND/84	NC	2.36
AB68	No	Spontaneous 166/88	**Gross lesions	300	0.049	1,689	20,059	NC	ND	***
AE62	No	Caesarean 55/30	Foetal death	NA	NA	NA	NA	7.51/29	4.51	8.07
AB18	Yes	Caesarean 159/128	No	480	0.034	1,514	30,208	ND/36	ND	ND
AA14	Yes	Caesarean 71/44	^#^Foetal death	NA	NA	NA	NA	ND/21, 33, 44	ND	ND
AB28	Yes	Caesarean 150/126	No	360	NC	NC	NC	ND/126	ND	ND
AD18	Yes	Spontaneous 145/92	No	340	NC	NC	2,034	ND/22	ND	ND
AG142	Yes	Caesarean 48/13	Foetal death	NA	NA	NA	NA	5.21/2 5.48/13	6.55	7.93

GA: gestational age; dpi: days post-inoculation; NA: not applicable; NC: not collected; ND: RNA not detected by real-time polymerase chain reaction (RT-PCR) and viral recovery in neonate Swiss mice; PRNT: plaque reduction neutralisation testing; #accidental foetal death; *birth weight in grams; **lissencephaly and ventriculomegaly; ***ZIKV recovered after Swiss mice intracerebral inoculation.

More than 50 ZIKV vaccine candidates are now in different preclinical and clinical phases.^88^ ^,^ ^89^ ^,^ ^90^ Proof-of-concept studies in mice and marmosets^91^ demonstrated a protective effect against ZIKV-induced teratogenesis during pregnancy.

Promising phase-1 clinical trials have evaluated the safety and efficacy of candidate anti-Zika vaccines. One of them was based on inactivated, viral vectors, and DNA vaccines. Immunogenicity ranged from 10% to 100% in geometric mean titre (GMT) (6.3; 95% confidence interval (CI): 3.7-10.8) observed among recipients of single-dose inactivated anti-Zika vaccine. For DNA vaccines, the seroconversion rate ranged from 60% to 100%, with the highest seroconversion rate (100%) and GMT (2871; 95% CI: 705.3-11688). For the viral vector vaccine (Ad26.ZIKV.001), the seroconversion rate was 100%, and GMT peaked after two shots with both low and high-dose vaccines.^92^

Despite worldwide efforts, no effective vaccine or monoclonal antibodies against ZIKV were licensed for human use until May 2024. Unfortunately, ZIKV continues to circulate low in some areas and can re-emerge in naïve populations.^90^ ^,^ ^93^ Public education initiatives targeting insecticide applications and innovative approaches, for example, manipulating vector bacterial symbionts such as Wolbachia, to combat mosquito transmission arboviruses need an approach integrating antiviral research, vaccination, and vector control.^85^ From 2015 to 2020, 3,591 cases of CZS were confirmed in Brazil, with an incidence of 44.03 cases per 1,000 live births and a specific mortality of 12.35 deaths per 1,000 live births.^94^ The World Health Organisation is alert to continued ZIKV vaccine development efforts. The initiative for Vaccine Research and the National Institutes of Health National Institute of Allergy and Infectious Diseases co-hosted a meeting of experts in March 2018 to identify strategies to demonstrate vaccine effectiveness given the worsening incidence of ZIKV disease.^95^ A wide variety of formulations are being studied, including live virus vaccines, inactivated vaccines, whole-virus vaccines, subunit vaccines, mRNA, DNA, protein, and vector-based platforms.^88^ In a novel live-attenuated ZIKV strain (titled Z7) generated by inserting 50 RNA nucleotides (nt) into the 5′ untranslated region (UTR) of the ZIKV Cambodian strain (FSS13025), the neurovirulence, immune antagonism, and mosquito infectivity of Z7 were attenuated compared with those of other isolates. Z7 induced robust humoral and cellular immune responses that completely prevented viremia after infection with the ZIKV strain PRVABC59 in type I IFN-deficient (Ifnar1−/−) mice; however, additional studies confirming the prevention of ZIKV-induced teratogenesis need to be performed in new preclinical studies.^96^

In conclusion

The results of the present study indicate that ZIKV replicates in trophoblast cells, transposes the placental barrier, and induces inflammation. Subsequently, ZIKV quickly reaches foetal blood, crosses the foetal haematoencephalic barrier (neuroinvasion), replicates in foetal NPCs, and induces neuroinflammation, causing CZS, foetal death and death. New preventive and therapeutic approaches to prevent foetal ZIKV infections remain necessary since no licenced product is available until we finish this review.
